# Digital image processing method for estimating leaf length and width tested using kiwifruit leaves (*Actinidia chinensis* Planch)

**DOI:** 10.1371/journal.pone.0235499

**Published:** 2020-07-06

**Authors:** Wanhong Zhang

**Affiliations:** Institute of Soil and Water Conservation, Northwest A & F University, Yangling, China; Newcastle University, UNITED KINGDOM

## Abstract

In this study, an image-analysis-based method is proposed to estimate the length and width of plant leaves, some of which have concave tips and petiole insertions. Here, kiwifruit leaves are used. Using the proposed method, orthophotographs of the leaves were obtained through image segmentation and image correction. The coordinates of the pixels along the edge of the kiwifruit leaf were then extracted. Finally, the leaf length was determined based on the sum of the number of pixels between the leaf tip and petiole insertion, and the leaf width was calculated based on the sum of the number of pixels for the widest region of the leaf. The experimental results were evaluated by analyzing the mean absolute error (MAE), root mean square error (RMSE), and accuracy. The maximum MAE and RMSE for the three examined cultivars are 0.281 and 0.366 cm, respectively. The analysis also showed that the proposed method outperforms other prevalent approaches, achieving high accuracy rates of 98.88% and 98.83% for the length and width of kiwifruit leaves, respectively. The low MAE and RMSE and high accuracy prove the capability of the proposed method with regard to calculating the length and width of such leaves. In addition, the proposed method can be extended to other plants whose leaf shape is comparable to that of a kiwifruit leaf.

## Introduction

The surface area of a plant leaf is a crucial parameter because it influences the interception of radiation, water and energy exchange processes, and crop growth and productivity [1]. Several researchers have therefore aimed to precisely measure leaf areas, which have traditionally been estimated quantitatively through both direct and indirect methods [2]. Direct methods, including the use of a leaf area meter, scanner, or blueprinting, involve destructive plant sampling. It is thus impossible to perform successive measurements of the same leaf. In addition, measuring the leaf area using a direct method is labor intensive [3]. By contrast, an alternative nondestructive and indirect method is the use of a regression model applied between the area of the leaf and one or more of its dimensions (length and/or width); this permits the repeated sampling of the same leaves, and is inexpensive [4, 5]. Using this approach, agronomists and physiologists have estimated the leaf areas of several plants, including chestnut [6], hazelnut [5], fava bean [7], ginger [8], maize [9], onion [10], pepper [4], and cacao [11]. The establishment of the regression models used to quantify the relationship between the surface areas and dimensions of leaves requires numerous data points consisting of the lengths and widths of the leaves collected from different levels of the canopy [12]. The leaf length and width have generally been measured using a ruler [3, 4, 10, 13–15]. Although useful, this method is laborious and can occasionally cause significant errors. Some studies have also used a leaf area meter, such as an LI-3000C (LICOR Biosciences, Lincoln, Nebraska, USA), to measure the length and width of plant leaves. A leaf area meter provides immediate, accurate, and portable measurements of the plant leaf length and width [16]. Nevertheless, such devices are expensive and perform poorly when measuring the area of exceptionally wide leaves [17].

Therefore, for an accurate monitoring of leaf growth and the development of a reasonable regression model for estimating the plant leaf area, an appropriate method for measuring the plant leaf length and width is necessary. Digital image processing methods have recently been proven to be reliable and cost-effective tools for use in various fields of agriculture [16, 18]. For example, digital image processing software, such as ImageJ, LAMINA, Macf-IJ, and Lamina2shape, have been developed to measure leaf dimensions [16, 18, 19]. Methods based on image processing have also been proposed to classify plant leaves and describe their morphology [20–23]. Depending on the software and method used, leaf measurements may be satisfactory for plant leaves with convex leaf tips and petiole insertions. When measuring the length of leaves with concave tips or petiole insertions, however, existing methods and software often provide poor measurement results because existing image processing methods for measuring the dimensions of plant leaves have mainly been developed based on the shapes of leaves with convex tips and petiole insertions. These methods are therefore unsuitable for measuring the dimensions, particularly the length, of leaves with concave tips and petiole insertions. In addition, when measuring the leaf length using digital image processing methods, the leaf width is frequently expressed as the sum of the number of pixels in the longest row in the binary image. As a result, tilting the leaf with respect to a vertical line can result in a large deviation of this sum from its true value. Furthermore, most of the proposed methods currently ignore the tilt relative to the vertical line.

Given the limitations outlined above, in this study, an appropriate method for measuring the leaf length and width was developed; the developed method is based on accurately locating the leaf tip and petiole insertion and on effectively removing the distortion and tilt of the image. Kiwifruit (*Actinidia chinensis* (Planch.)) leaves, the tips and petiole insertions of which are occasionally concave, are taken as an example.

## Materials and methods

### Experimental site

The experiment was conducted in a kiwifruit orchard located in Fufeng County, Shaanxi Province, China (latitude, 34°25′E; longitude, 107°53′N; altitude, 610 m). The county has loessial soil and semi-humid continental climate. The annual mean temperature is 12.4°C, the frost-free period is 209 days, the period of annual sunshine is 2,134 h, and the annual mean rainfall is 592 mm.

### Data collection

**Fig 1:**
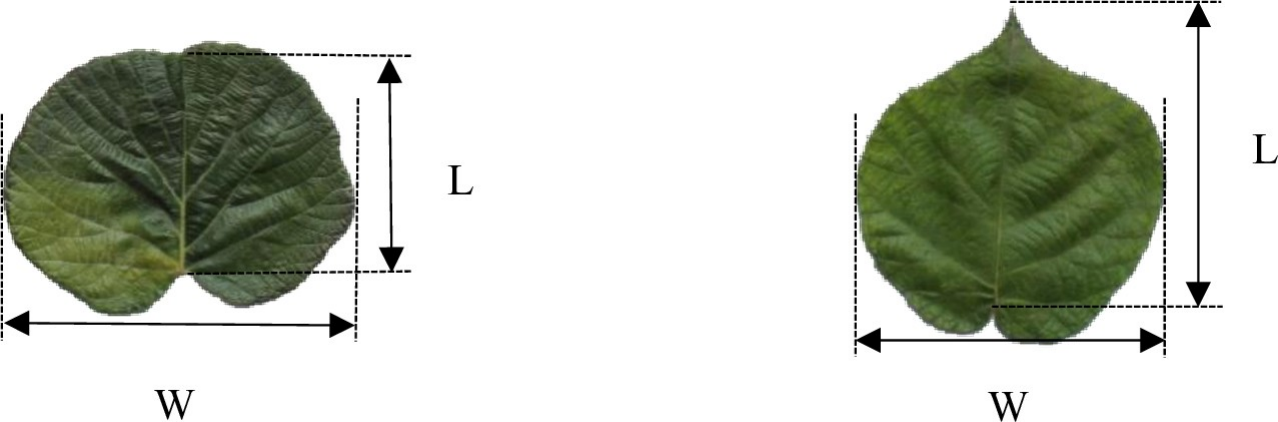
Diagram showing the position of the length and width measurements for kiwifruit.

This study was conducted on a private orchard, with permission from the owner. Three kiwifruit cultivars were selected for this research: Xuxiang (*A*. *chinensis* Planch), Hongyang (*A*. *chinensis* Planch), and Yate (*A*. *chinensis* Planch). This group of cultivars includes various leaf shapes that generally represent most of the kiwifruit leaf shapes available at the study site. For each cultivar, fully expanded leaves of different sizes were sampled at random from different levels in the canopy when the kiwifruits reached maturity to achieve a wide morphological variation in the sampling. The fresh leaves were carefully detached and placed in plastic bags and then taken directly to the laboratory for subsequent measurements of the length (L) and width (W) based on the definitions of plant leaf length and width, as described in [24] (Fig 1). In this study, we used manual measurements of the kiwifruit leaf length and width as the ground truth.

**Fig 2:**
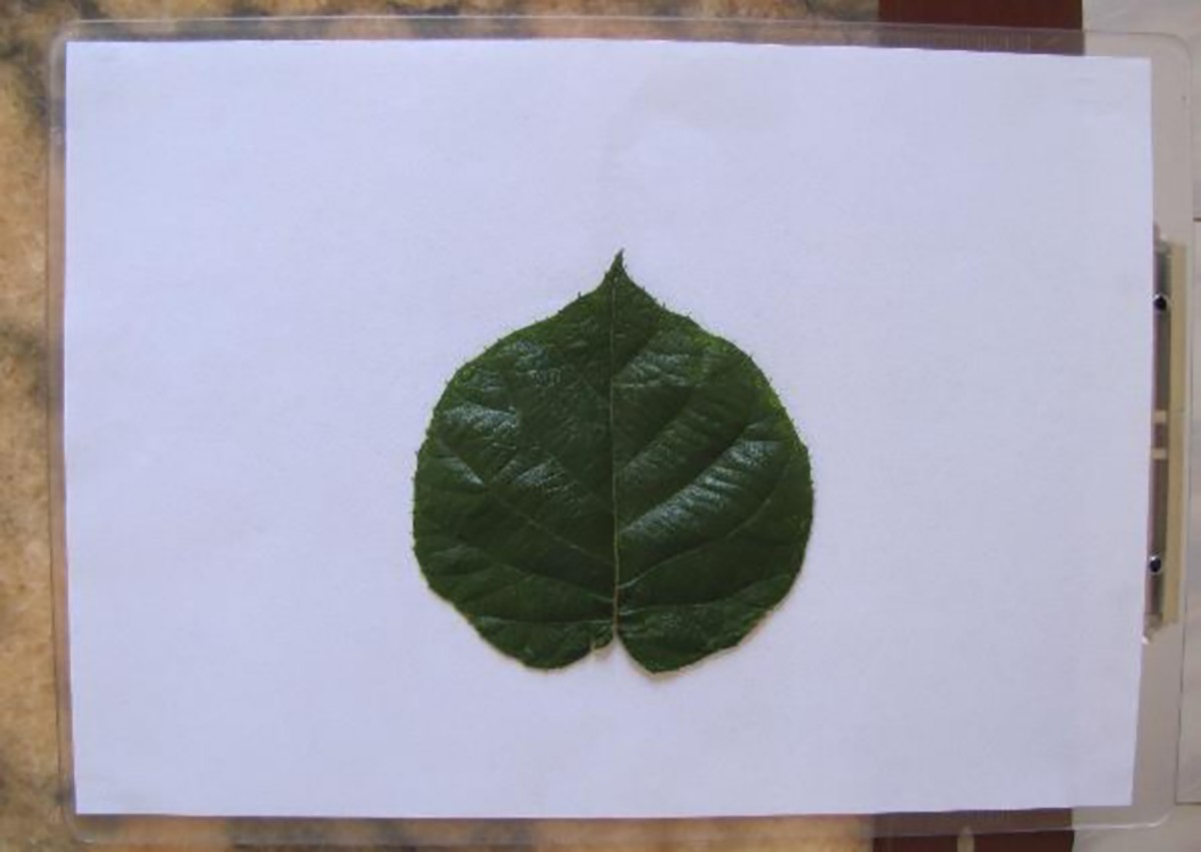
Example of a collected image.

Measurements were conducted based on image processing using MATLAB version R2015b (MathWorks, Inc., Natick, USA). Prior to image processing, leaf images were captured using a digital camera (Canon 500D with a resolution of 15.1 megapixels; Canon, Tokyo) by placing the samples against a fixed white background (21 cm × 29 cm). The captured leaf images were stored in a computer in the RGB color mode, as shown in Fig 2.

### Proposed method

**Fig 3:**
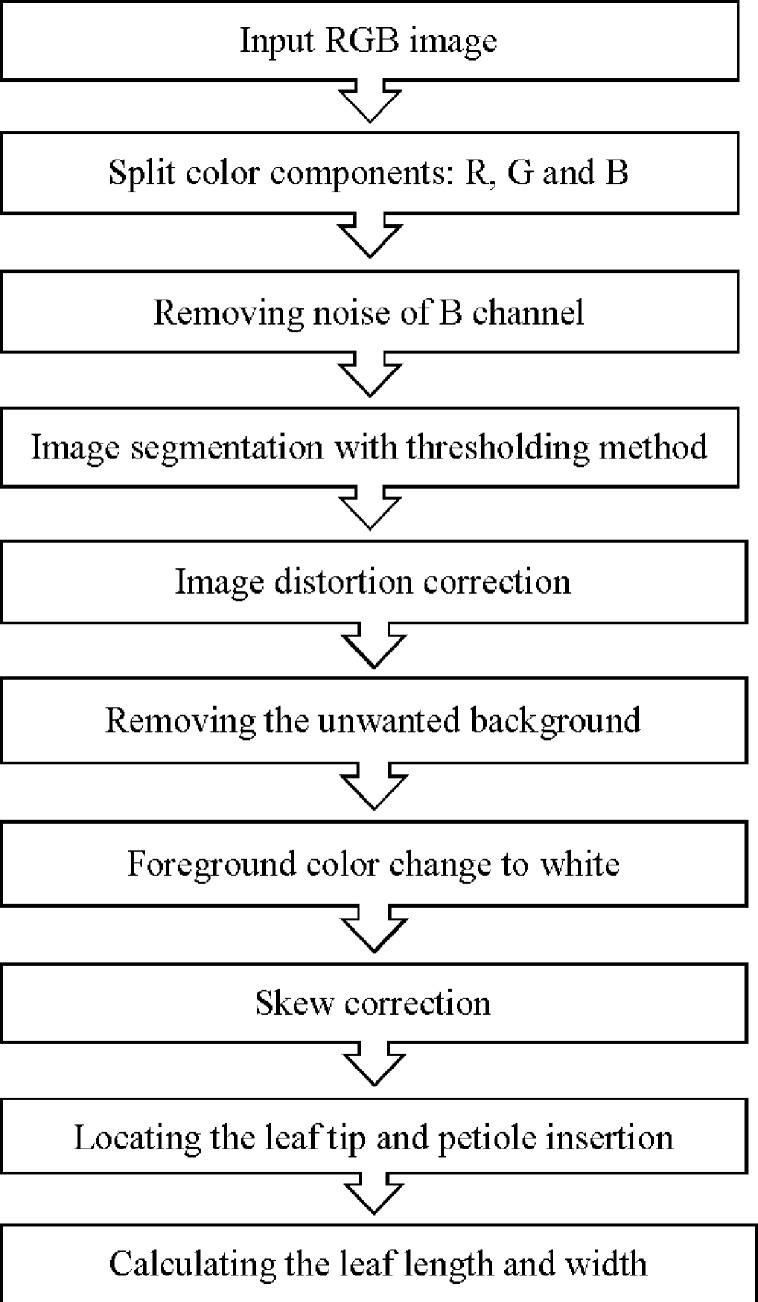
Flowchart of image processing steps for calculating kiwifruit leaf length and width.

The proposed method includes ten image processing steps, which are shown in the algorithm flowchart (Fig 3).

#### Image segmentation

In this study, we found a substantial difference in the grayscale contrast intensity between the white background and kiwifruit leaf in the B channel of the RGB (red, green, blue) color image. We thus used the thresholding method to segment the kiwifruit leaf from the background. The detailed procedure of the image segmentation is as follows.

**Fig 4:**
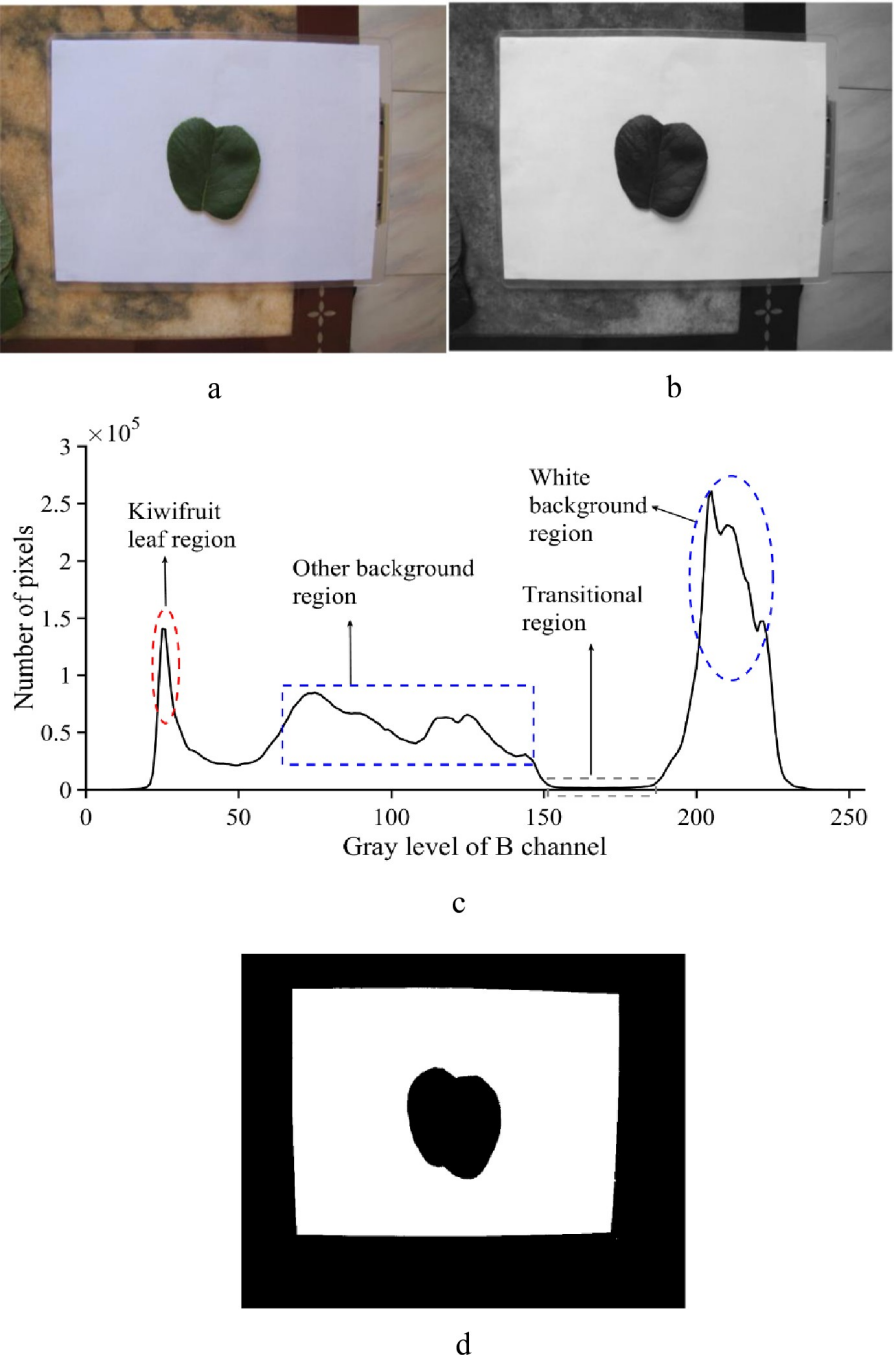
Segmenting the kiwifruit leaf from the background. (a) original image, (b) median filter denoising of the B-channel image, (c) histogram of the B-channel image, and (d) binary image.

We first acquired a matrix of pixels of the RGB image through image reading (Fig 4A). The color image was then divided into the R, G, and B color channels. Only the B color channel image was retained as the candidate image for the subsequent image segmentation. Because noise produced by the camera, an uneven illumination, and/or the image transmission have an impact on the image segmentation, it is necessary to remove such noise prior to the image segmentation. In this study, a 3 × 3 median filter was used for noise removal, a resulting image of which is shown in Fig 4B. Thereafter, a histogram based on the B color channel was drawn, and the valley located at the right end of the histogram was chosen as the threshold (Fig 4C). Finally, the kiwifruit leaf was segmented from the background using the acquired threshold (Fig 4D).

#### Image distortion correction and skew correction

**Fig 5:**
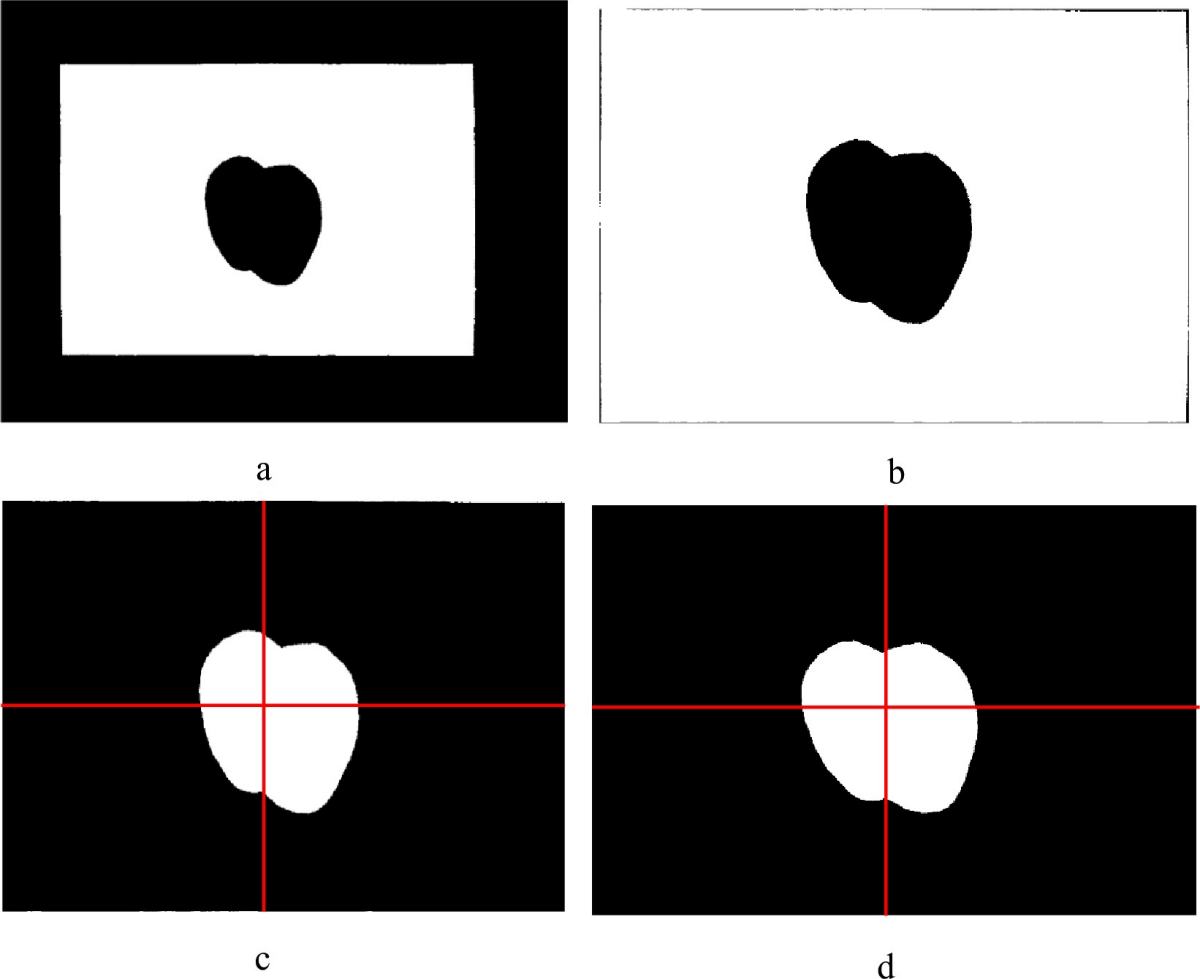
Image correction. (a) image after implementing geometrical distortion correction, (b) image after removing unwanted background, (c) tilt of kiwifruit leaf with respect to the vertical line (the red perpendicular lines are the reference lines), and (d) image after performing skew correction.

Given that the optical distortion generated by the imaging system lenses and the perspective distortion created by the viewing angle have a negative effect on the measurement accuracy of the kiwifruit leaf dimensions, the binary image was corrected before the leaf length and width were measured using the method proposed in [25]. A corrected image is shown in Fig 5A.

After correcting the geometrical distortion of the image, we implemented the skew correction of the foreground. To reduce the computational cost, the unwanted background was first removed from the corrected images. This was performed based on a bounding box enclosing the rectangle of the white background, as shown in Fig 5B. Then, the background color and kiwifruit leaf color were swapped, and some pixels belonging to the background were removed (Fig 5C). Observation shows that the central vein tends to become straight when it approaches the leaf base (Fig 4A). To calculate the slope of the central leaf vein near the leaf base, we thus extracted the coordinates of two different points from the region of the central vein where a straight line was present. The angle of inclination of the foreground was calculated in terms of the slope, and a skew correction was applied using image rotation algorithms (Fig 5D). The corrected image was utilized in the measurement of the length and width of the kiwifruit leaf.

#### Locating the kiwifruit leaf tip and petiole insertion

The determination of the plant leaf length requires accurately locating the position of the leaf tip and petiole insertion when gauging the length of the kiwifruit leaf. For this reason, the coordinates of each pixel of the leaf edge were obtained to help locate the positions of the leaf tip and petiole insertion. A close observation revealed that the leaf tip and petiole insertion of the kiwifruit may be classified as either concave or convex. We thus developed two different methods for locating the position of the leaf tip and petiole insertion according to the shape features of the kiwifruit leaf.

**Fig 6:**
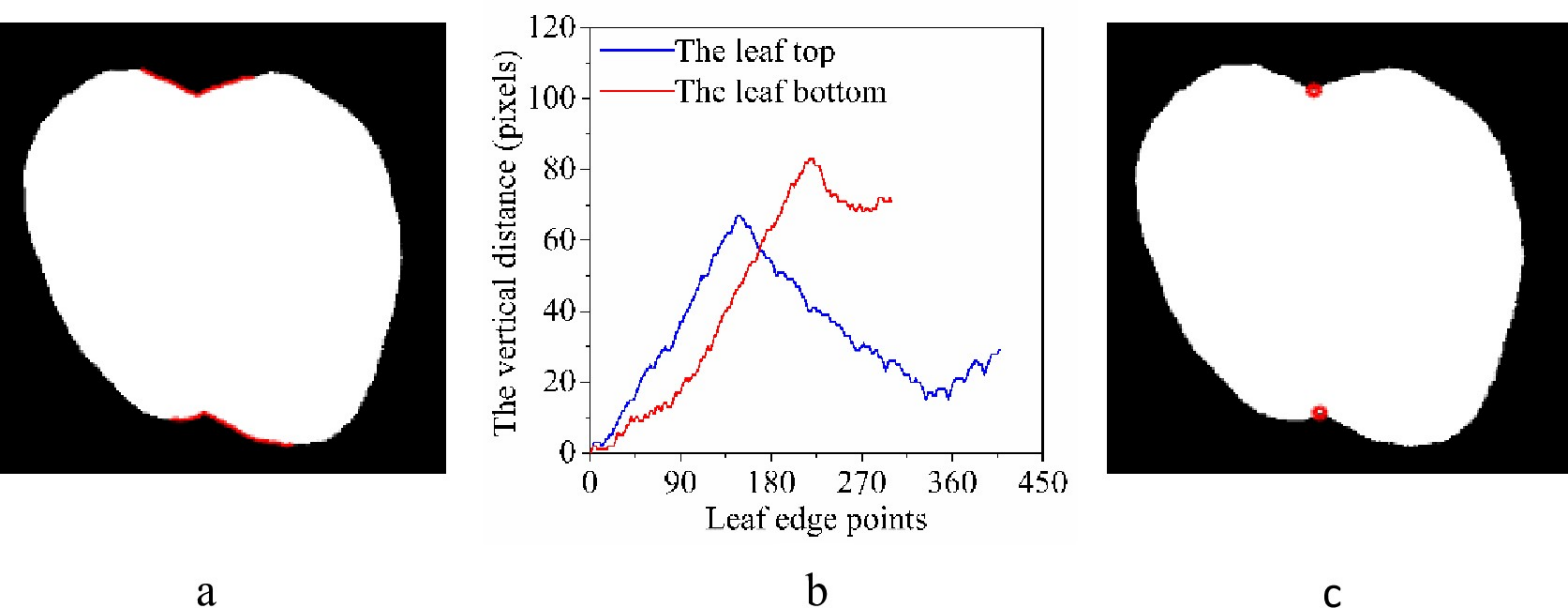
Locating the concave leaf tip and petiole insertion of a kiwifruit leaf. (a) the red region of the leaf edge represents the selected region, (b) the solid blue line represents the vertical coordinate differences between the pixel points at the top of the leaf edge and the highest pixel point, and the solid red line represents the vertical coordinate differences between the pixel points at the bottom of the leaf edge and the lowest pixel point, and (c) the red circles indicate the position of the leaf tip and petiole insertion.

When detecting the location of a concave leaf tip or petiole insertion, it was impossible to directly locate them by relying on the global extremum owing to the relatively complicated structure of the tip and petiole insertion, but it was feasible to position the target points by searching for the local extrema. In this case, choosing the appropriate region of the leaf edge played an important role in successfully implementing the location identification. In this study, the rules for determining the region of the leaf edge were as follows: First, the position of the leaf tip and petiole insertion must be inside the chosen region. Second, the scope of the chosen region must be as small as possible to minimize the number of computations and reduce interference from the other edge pixels. Because a segment of the leaf edge may be detected using two endpoints, the proposed method determines the endpoints of the leaf edge segment by searching for two feature points of the leaf edge based on the shape features of the kiwifruit leaf. The top-left or top-right area of each kiwifruit leaf with a concave tip consistently contained the highest point of the leaf (Fig 4A); such points may be easily located by searching for the global extrema. Therefore, the point located at the top-left or top-right of a kiwifruit leaf was chosen as a single endpoint. In addition, a part of the top of the kiwifruit leaf tends to be flat; the second endpoint can then be determined by searching for the most frequently occurring coordinates in a leaf edge region. For the petiole insertion, a method similar to that described above was used for the bottom of the leaf. Fig 6A shows the selected areas at the top and bottom of the kiwifruit leaf edge.

After determining the appropriate kiwifruit leaf edge region, the difference between the vertical (Y) coordinate of each pixel within the chosen region of the leaf edge at the top of kiwifruit leaf and the vertical coordinate of the actual highest point of the kiwifruit leaf was obtained. Similarly, the difference between the vertical coordinate of each pixel within the chosen region of the leaf edge at the bottom of the kiwifruit leaf and the vertical coordinate of the actual lowest point of the kiwifruit leaf was also obtained. The corresponding curve was then plotted (Fig 6B). The leaf tip and petiole insertion were located using an algorithm to find the peak value of the curve (Fig 6C).

**Fig 7:**
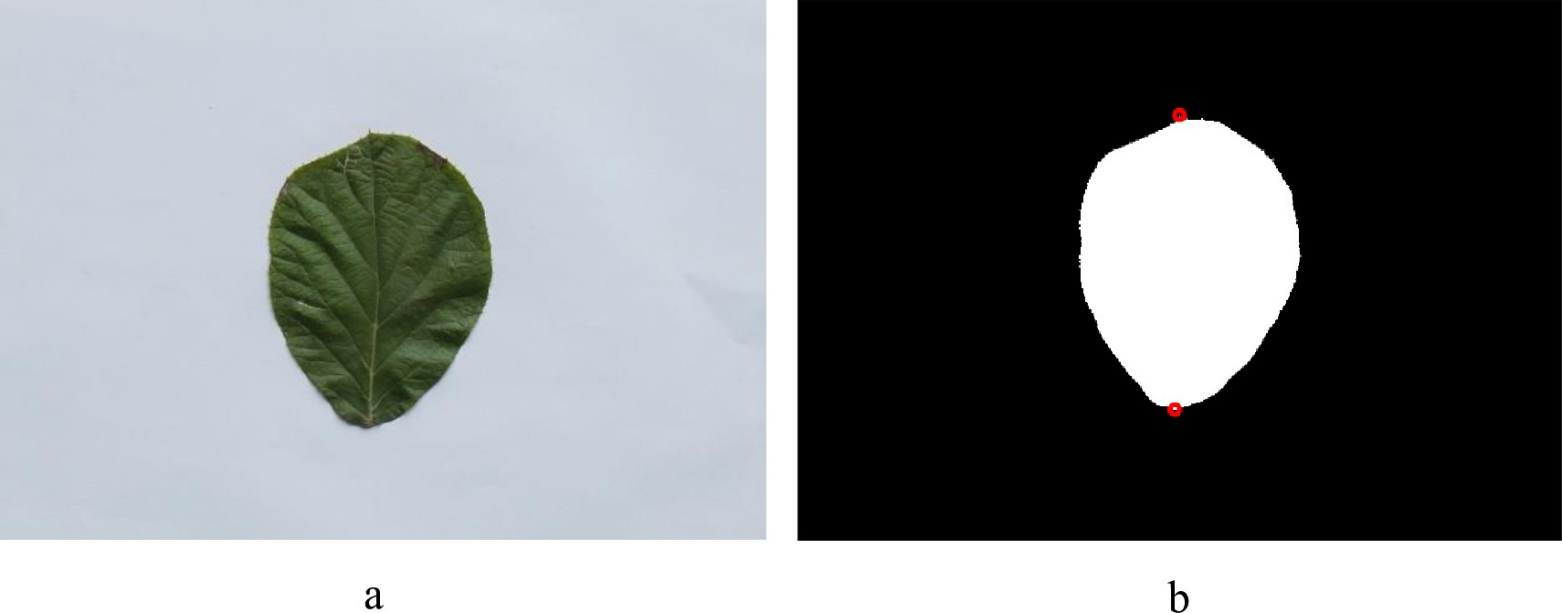
Locating the convex leaf tip and petiole insertion of the kiwifruit leaf. (a) original leaf and (b) determined leaf tip and petiole insertion shown in the red circles.

Regarding convex kiwifruit leaf tips and petiole insertions, their positions were identified by searching for the global minimum and maximum coordinates of the leaf edge pixels. In this way, the positions of the convex kiwifruit leaf tips and petiole insertions can be conveniently determined, as shown in Fig 7.

### Measurement of kiwifruit leaf length and width

The length of the kiwifruit leaf (pixels) was acquired using the two-point distance formula given in Eq (1). The width of the kiwifruit leaf (pixels) was measured in terms of the number of pixels between the two edge points where the lamina is the widest. After acquiring the leaf length and width in pixels, the pixel values were converted into real values based on the number of pixels per centimeter, which was derived from the ratio between the number of pixels spanning the white background and its actual size:

$$D=\sqrt{{\left(a_{i}-b_{i}\right)}^{2}-{\left(a_{j}-b_{j}\right)}^{2}},$$ (1)

where *D* is the distance between points *a* and *b*, *a*_*i*_ and *b*_*i*_ are the abscissa of points *a* and *b*, and *a*_*j*_ and *b*_*j*_ are the ordinates of points *a* and *b*, respectively.

### Validation of calculation results

To evaluate the reliability of the methods proposed herein, we used a ruler to measure the length and width of 117 kiwifruit leaf samples, each of which was measured three times by three different people. Based on manual measurements and the values calculated using the proposed method, the RMSE and MAE of the leaf length and width were calculated using Eq (2) and (3), respectively. Subsequently, taking the average of the manual measurements as a benchmark, the accuracy of the proposed method was compared with those of ImageJ version 1.52a (National Institute of Health, USA), Digimizer version 5.4.3 (MedCalc Software Ltd., Belgium) and the method proposed by Ali et al. [26]. The accuracy is formulated as Eq (4). Next, to determine whether there are inconsistencies between the calculated and measured values, a scatter plot of the calculated versus measured values was produced with a reference line at y = x. In this study, data analysis was conducted using Excel 2019 (Microsoft Inc., USA).

$$RMSE=\sqrt{\frac{\sum_{i=1}^{n}{\left(x_{i}-y_{i}\right)}^{2}}{n-1}},$$ (2)

$$MAE=\sum_{i=1}^{n}\left|\frac{x_{i}-y_{i}}{n}\right|,$$ (3)

$$Accuracy\;rate=\left(1-|\frac{y_{i}-x_{i}}{x_{i}}|\right)\times 100\%,$$ (4)

where *x*_*i*_ and *y*_*i*_ are the measured and calculated leaf length or width for the ith evaluation, respectively, and *n* is the number of samples.

## Results

**Fig 8:**
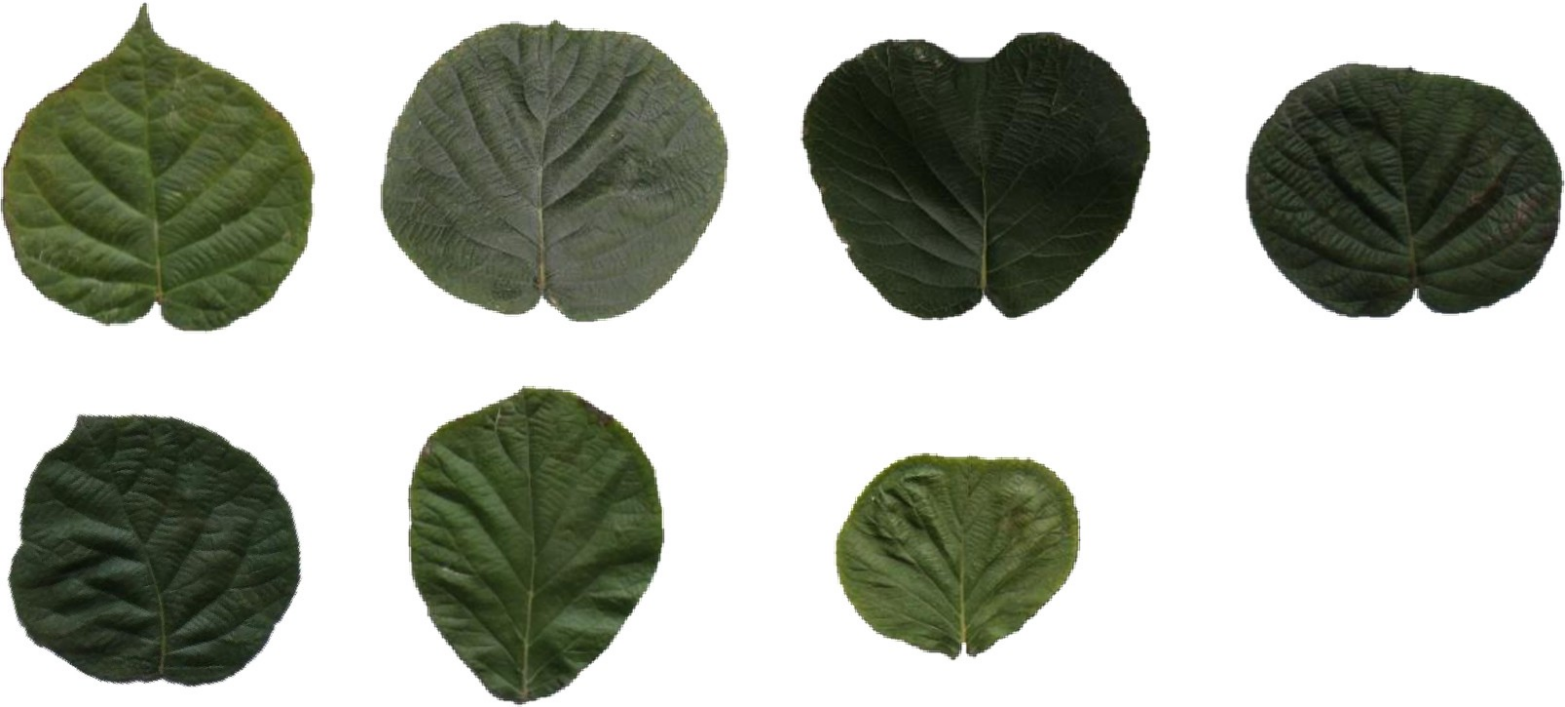
Self-collected dataset of kiwi fruit leaf images.

**Fig 9:**
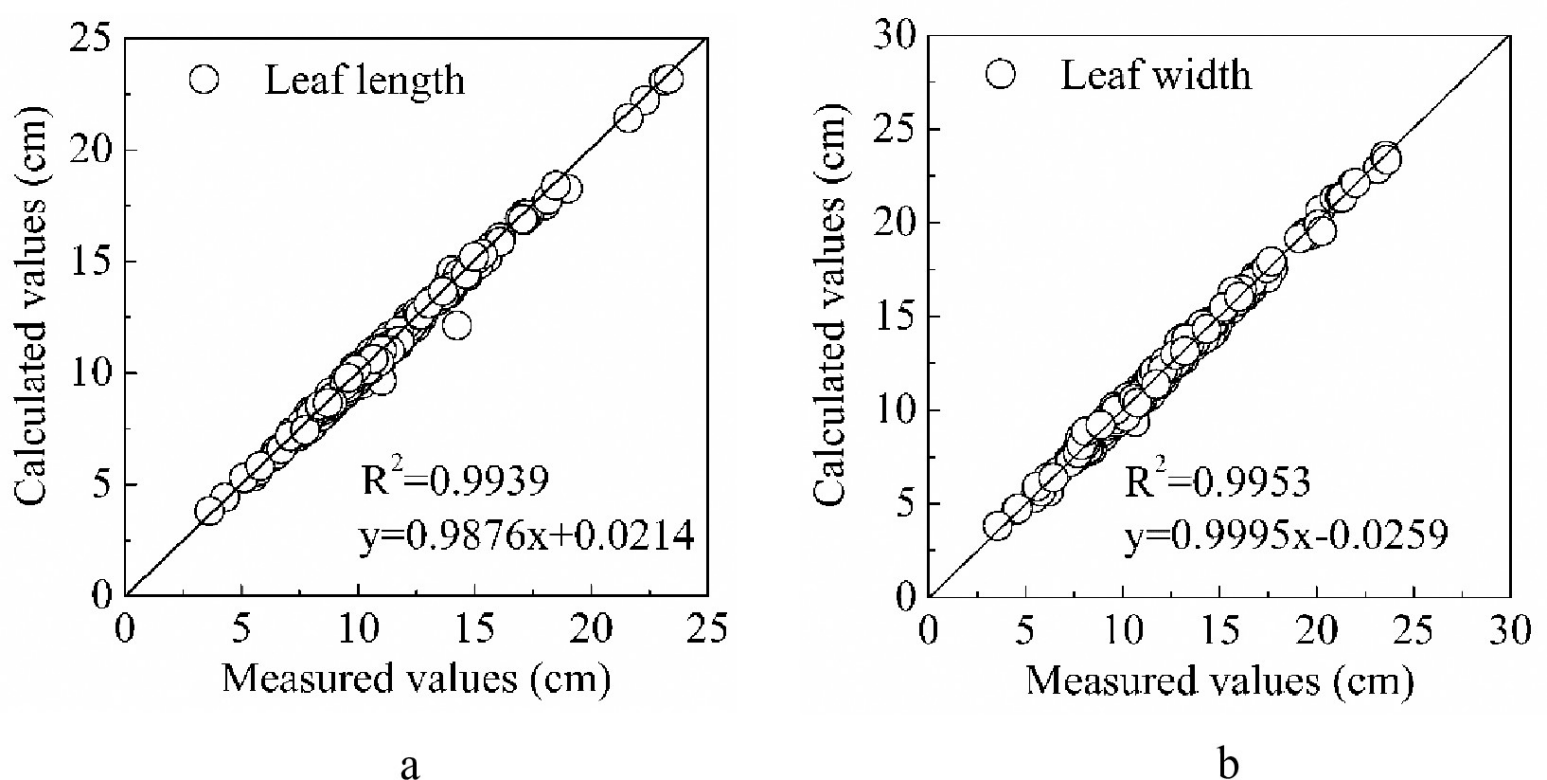
Dispersion of data between the measured and calculated values of the leaf length and width. (a) leaf length and (b) leaf width, where the solid line is the y = x line.

Different types of kiwifruit leaves collected from three kiwifruit cultivars were used to test the performance of the proposed method (Fig 8). It was found that the length of the kiwifruit leaves ranged from 3.6 to 23.3 cm, with an average value of 10.3 cm, whereas the width of the kiwifruit leaves varied from 3.5 to 23.6 cm, with an average value of 12.0 cm. Comparisons between the measured and calculated length and width of kiwifruit leaves are illustrated in Fig 9. The coefficient of determination (R^2^) values for the leaf length and width are 0.993 and 0.995, respectively, indicating that the calculated results are acceptable [27]. In addition, the calculated and measured values closely follow the y = x line, which suggests a reliable relationship between the measured and calculated leaf length and width for all three kiwifruit cultivars chosen in this experiment.

To evaluate the proposed approach quantitatively, three evaluation indexes, namely, the MAE, RMSE, and accuracy, were used. The calculated results are presented in Tables 1 and 2.

**Table 1:** MAE and RMSE for length and width of kiwifruit leaves for the three cultivars examined.

Leaf dimension	A. *chinensis* Planch Xuxiang		*A*. *chinensis* Planch Hongyang		A. *chinensis* Planch Yate	
	MAE	RMSE(cm)	MAE	RMSE (cm)	MAE	RMSE (cm)
Length	0.224	0.302	0.188	0.259	0.165	0.227
Width	0.248	0.337	0.281	0.366	0.258	0.349

**Table 2:** Comparison of the accuracy between the proposed and other popular methods.

Dimension	Methods	Accuracy rate (%)
Length	Digimizer	79.55
	Ali, et al 2012	82.15
	ImageJ	88.90
	Proposed method	98.88
Width	Digimizer	94.15
	Ali, et al 2012	93.69
	ImageJ	97.33
	Proposed method	98.83

According to Table 1, the MAE of the leaf length was 0.165, 0.188, and 0.224 for the Yate, Hongyang, and Xuxiang cultivars, respectively. The RMSE of the leaf length was 0.227, 0.259, and 0.302 cm, for Yate, Hongyang, and Xuxiang, respectively. Compared with the leaf length, the MAE and RMSE for the leaf width have slightly higher values for all cultivars. As indicated in Table 1, the RMSE of the leaf width was 0.337 cm for Xuxiang, 0.349 cm for Yate, and 0.366 cm for Hongyang, and the MAE was 0.248 for Xuxiang, 0.258 for Yate, and 0.281 for Hongyang. Among all three cultivars, Hongyang had the highest RMSE and MAE, whereas Yate had the lowest.

Table 2 shows that when measuring the kiwifruit leaf width, the proposed method produced an accuracy of 98.83%, followed by 97.33% for ImageJ, 94.15% for Digimizer, and 93.69% for the method proposed by Ali et al. (2012). Table 2 also indicates that, when calculating the length of the kiwifruit leaf, the proposed method performed well, with a high accuracy of 98.88%; the performance of the other three methods was poor, with ImageJ being slightly better among them. As a further observation, the results calculated by all four methods for the width of the kiwifruit leaf were superior to those for the length.

## Discussion

When using a thresholding method, it is crucial to choose a suitable threshold for separating one object from another in an RGB image. In previous studies, the threshold value was frequently determined directly based on the color features of the foreground in an RGB image when an obvious difference in color occurred between the foreground and background; alternatively a suitable threshold may be obtained after converting the RGB color space into HSV, L a* b*, or other color coordinates [28, 29]. In this study, it was determined that the surface color of the kiwifruit leaf was not uniform, as shown in Fig 2. This may interfere with the determination of an appropriate threshold value based on an RGB image of a kiwifruit leaf. This in turn constrains the image segmentation when applying the thresholding method. It was also found that there was little color variation within the region of the white paper (Fig 2). In this context, we obtained a threshold value according to information from the white paper in the RGB image and successfully separated the kiwifruit leaf from the background using the threshold value chosen from the target image, as illustrated in Fig 4D. In contrast with the commonly used thresholding method that often acquires a threshold value from the foreground, the proposed method simplifies the procedure of producing an appropriate threshold and is easy to apply in practice. In this study, a standard A4 sized sheet of white paper was chosen as the background, and the pixel coordinates of its four vertices could be easily detected in a binary image using an image processing approach. This facilitated the correction of any geometrical distortion of the target image by rearranging the pixel coordinates of the four vertices of the white rectangular paper. In addition, the true value of each pixel could be determined by reference to the known paper size. In this way, the pixel values obtained from the target image along the length and width of the kiwifruit leaf could be easily transformed into the actual length according to the number of pixels in one centimeter of standard A4 paper, whose actual length is known.

The length and width of the kiwifruit leaf were estimated based on image processing. To demonstrate the performance of the proposed algorithm, the calculated results were evaluated by calculating the MAE and RMSE and comparing the accuracy of the proposed method with the results produced by ImageJ, Digimizer, and Ali et al.’s approach. The maximal MAE (0.281) and RMSE (0.366 cm) in Table 1 show that the proposed algorithm is capable of accurately calculating the length and width of the kiwifruit leaf. As shown in Table 2, the proposed method also outperforms ImageJ, Digimizer, and the method developed by Ali et al. (2012) with a high accuracy. In addition, compared with the measurements of the leaf width, the measurements of the kiwifruit leaf length performed using ImageJ, Digimizer, and Ali et al.’s method exhibited a larger deviation from the true values for the following reasons. First, the segmentation method used by the proposed method produces good results, which means that the loss of the leaf edge pixels, which often results from a poor segmentation, may be minimized when extracting the leaf edge from the images. Thus, the structure of the leaf edge is well preserved by the proposed method. By contrast, the other three methods poorly segmented the kiwifruit leaf from the background, although ImageJ produced a relatively better segmentation result than Digimizer or Ali et al.’s method. Second, the proposed method not only rectified the geometrical distortion of the digital images but also corrected for the tilt of the kiwifruit leaf with respect to a vertical line. As a result, the positions of the kiwifruit leaf tip and petiole insertion could be accurately located, and the sum of the number of pixels belonging to the longest row in the binary image was extremely close to the corresponding true value after the image correction. In contrast to the proposed method, ImageJ, Digimizer, and Ali et al.’s method do not perform image correction when measuring the dimensions of the plant leaf. Furthermore, ImageJ, Digimizer, and Ali et al.’s method regard the maximum length of the kiwifruit leaf as equivalent to the leaf length, but this is only appropriate when measuring the length of a leaf with a convex leaf tip and convex petiole insertion. In the case of a kiwifruit leaf with a concave leaf tip and concave petiole insertion, the maximum leaf length often deviates substantially from the distance from the leaf tip to the petiole insertion. A careful observation in the kiwifruit orchard also revealed that the leaf tip and petiole insertion of many kiwifruit leaves have a concave indentation. Large errors thus occur when calculating the length of the kiwifruit leaves using ImageJ, Digimizer, or the method proposed by Ali et al. (2012).

There is currently a need for research on estimating leaf areas using nondestructive methods to preserve plant integrity. One of the most widely used nondestructive methods in this regard is the assessment of the leaf area through mathematical equations based on linear measurements such as measurements of the leaf length and width [15]. Consequently, to establish reasonable mathematical equations, it is necessary to acquire accurate length and width data for plant leaves. The results of this study confirm that the proposed methodology can accurately compute the length and width of kiwifruit leaves and help researchers in utilizing mathematical models in this regard. The proposed method is also feasible for validating the accuracy of leaf area meters before using them to measure the area of kiwifruit leaves.

## Conclusions

Images of kiwifruit leaves were captured using a consumer-grade RGB camera under specific background conditions. An algorithm for calculating the length and width of the kiwifruit leaves was developed based on image processing. We found that the threshold values derived from the background (white paper) are reasonable, and an appropriate segmentation of the background and kiwifruit leaf was obtained by applying the selected threshold values using a thresholding method for an RGB image. Our results also demonstrate that the proposed method accurately located the tip and petiole insertion of a kiwifruit leaf, making it possible to accurately calculate the length. In addition, the method performed well in determining the leaf width. Furthermore, the proposed method can be extended to leaves of plants with a leaf shape comparable to that of a kiwifruit leaf.

## Supporting information

S1 Appendix Code used for image segmentation. (PDF)

S2 Appendix Code used for correcting the geometrical distortion of the image. (PDF)

S3 Appendix Code used for correcting the skew of the image. (PDF)

S4 Appendix Code used for locating the concave leaf tip and petiole insertion of the kiwifruit. (PDF)

S5 Appendix Code used for calculating the length and width of a kiwifruit leaf. (PDF)
