# Supplementary material for: Digital image processing method for estimating leaf length and width tested using kiwifruit leaves (Actinidia chinensis Planch)
Source: PLoS One. 2020 Jul 6;15(7):e0235499. doi: 10.1371/journal.pone.0235499 (PMC7337316; doi:10.1371/journal.pone.0235499)
Supplement: S1 Appendix — (PDF) [file pone.0235499.s001.pdf]

Image segmentation

```
>> I=imread('sample.jpg');
>> R=I(:,:,1);
>> G=I(:,:,2);
>> B=I(:,:,3);
>> B1=medfilt2(B);
>> figure,imhist(B1);
>> bw=B1>=170;
>> bw1=bwareaopen(bw,round(0.20*sum(sum(bw))));
>> figure,imshow(bw1);
```
