# Supplementary material for: Digital image processing method for estimating leaf length and width tested using kiwifruit leaves (Actinidia chinensis Planch)
Source: PLoS One. 2020 Jul 6;15(7):e0235499. doi: 10.1371/journal.pone.0235499 (PMC7337316; doi:10.1371/journal.pone.0235499)
Supplement: S2 Appendix — (PDF) [file pone.0235499.s002.pdf]

Geometrical distortion correction of image

```
cameraParams =
  cameraParameters with properties:

   Camera Intrinsics
                    IntrinsicMatrix: [3x3 double]
                        FocalLength: [3.3681e+03 3.3651e+03]
                     PrincipalPoint: [1.9702e+03 1.4414e+03]
                               Skew: 3.9474
   Lens Distortion
                   RadialDistortion: [-0.1921 0.1983]
               TangentialDistortion: [-4.2635e-04 -8.9685e-04]
   Camera Extrinsics
                   RotationMatrices: [3x3x15 double]
                 TranslationVectors: [15x3 double]
   Accuracy of Estimation
              MeanReprojectionError: 0.6799
                 ReprojectionErrors: [54x2x15 double]
                  ReprojectedPoints: [54x2x15 double]
   Calibration Settings
                        NumPatterns: 15
                        WorldPoints: [54x2 double]
                         WorldUnits: 'mm'
                       EstimateSkew: 1
    NumRadialDistortionCoefficients: 2
       EstimateTangentialDistortion: 1
>> bw2=undistortImage(bw1,cameraParams);
>> figure,imshow(bw2);
>> img=bw2;
>> [M N]=size(img);
>> dot=ginput();
>> w=round(sqrt((dot(1,1)-dot(2,1))^2+(dot(1,2)-dot(2,2))^2));
>>h=round(sqrt((dot(1,1)-dot(3,1))^2+(dot(1,2)-dot(3,2))^2));
>>y=[dot(1,1) dot(2,1) dot(3,1) dot(4,1)];
>>x=[dot(1,2) dot(2,2) dot(3,2) dot(4,2)];
>>Y=[dot(1,1) dot(1,1) dot(1,1)+h dot(1,1)+h];
>>X=[dot(1,2) dot(1,2)+w dot(1,2) dot(1,2)+w];
>>B=[X(1) Y(1) X(2) Y(2) X(3) Y(3) X(4) Y(4)]';
>>A=[x(1) y(1) 1 0 0 0 -X(1)*x(1) -X(1)*y(1);
      0 0 0 x(1) y(1) 1 -Y(1)*x(1) -Y(1)*y(1);
      x(2) y(2) 1 0 0 0 -X(2)*x(2) -X(2)*y(2);
      0 0 0 x(2) y(2) 1 -Y(2)*x(2) -Y(2)*y(2);
```

```
        x(3) y(3) 1 0 0 0 -X(3)*x(3) -X(3)*y(3);
        0 0 0 x(3) y(3) 1 -Y(3)*x(3) -Y(3)*y(3);
        x(4) y(4) 1 0 0 0 -X(4)*x(4) -X(4)*y(4);
        0 0 0 x(4) y(4) 1 -Y(4)*x(4) -Y(4)*y(4)];
>>fa=inv(A)*B;
>>a=fa(1);b=fa(2);c=fa(3);
>>d=fa(4);e=fa(5);f=fa(6);
>>g=fa(7);h=fa(8);
>>rot=[d e f;
        a b c;
        g h 1];
>>pix1=rot*[1 1 1]'/(g*1+h*1+1);
>>pix2=rot*[1 N 1]'/(g*1+h*N+1);
>>pix3=rot*[M 1 1]'/(g*M+h*1+1);
>>pix4=rot*[M N 1]'/(g*M+h*N+1);
>>height=round(max([pix1(1) pix2(1) pix3(1) pix4(1)])-min([pix1(1) pix2(1) pix3(1) pix4(1)]));
>>width=round(max([pix1(2) pix2(2) pix3(2) pix4(2)])-min([pix1(2) pix2(2) pix3(2) pix4(2)]));
>>imgn=zeros(height,width);
>>delta_y=round(abs(min([pix1(1) pix2(1) pix3(1) pix4(1)])));
>>delta_x=round(abs(min([pix1(2) pix2(2) pix3(2) pix4(2)])));
>>inv_rot=inv(rot);
   for i = 1-delta_y:height-delta_y
       for j = 1-delta_x:width-delta_x
            pix=inv_rot*[i j 1]';
            pix=inv([g*pix(1)-1 h*pix(1);g*pix(2) h*pix(2)-1])*[-pix(1) -pix(2)]';
            if pix(1)>=0.5 && pix(2)>=0.5 && pix(1)<=M && pix(2)<=N
                imgn(i+delta_y,j+delta_x)=img(round(pix(1)),round(pix(2)));
            end
       end
   end
>>figure,imshow(imgn);
```
