# Supplementary material for: Digital image processing method for estimating leaf length and width tested using kiwifruit leaves (Actinidia chinensis Planch)
Source: PLoS One. 2020 Jul 6;15(7):e0235499. doi: 10.1371/journal.pone.0235499 (PMC7337316; doi:10.1371/journal.pone.0235499)
Supplement: S3 Appendix — (PDF) [file pone.0235499.s003.pdf]

Skew correction of image

```
>> cc=regionprops(imgn,'BoundingBox');
>> sta=cc.BoundingBox;
>> bw3=imcrop(imgn,sta);
>> bw4=bwareaopen(~bw3,round(0.25*sum(sum(~bw3))));

>> cursor1
cursor1 =
        Target: [1x1 Image]
      Position: [1821 1678]
>> cursor2
cursor2 =
        Target: [1x1 Image]
      Position: [1811 1742]
>> aa=struct2cell(cursor1);
>> for i=1:length(aa);
        cur=aa{i};
   end
>> bb=struct2cell(cursor2);
>> for ii=1:length(bb);
        cur1=bb{ii};
   end
>> slope=(cur(2)-cur1(2))/(cur(1)-cur1(1));
>> angle=atan(slope)*(180/pi);
>> imrotate_angle=90+angle
>> bw5=imrotate(bw4,imrotate_angle,'bilinear','crop');
```
