# Supplementary material for: Digital image processing method for estimating leaf length and width tested using kiwifruit leaves (Actinidia chinensis Planch)
Source: PLoS One. 2020 Jul 6;15(7):e0235499. doi: 10.1371/journal.pone.0235499 (PMC7337316; doi:10.1371/journal.pone.0235499)
Supplement: S4 Appendix — (PDF) [file pone.0235499.s004.pdf]

Locating leaf tip and petiole insertion

```
>> boundary=bwboundaries(bw5,'noholes');
>> for k=1:length(boundary);
        bound=boundary{k};
   end
>> x=bound(:,2);
>> y=bound(:,1);
>> miny=min(y);
>> maxy=max(y);
>> maxx=max(x);
>> minx=min(x);
>> indminy=find(y==miny);
>> indmaxx=find(x==maxx);
>> freq_vale_top=mode(y(indminy:min(indmaxx)));
>> freq_ind_top=find(y==freq_vale_top);
>> figure,imshow(bw5),hold on;
>>plot(x(indminy:max(freq_ind_top)),y(indminy:max(freq_ind_top)),'r','linewidth',2);
>>dif_top=y(indminy:max(freq_ind_top))-ones((max(freq_ind_top)-indminy+1),1)*
  miny;
>> dif_top_max=max(dif_top);
>> dif_top_max_ind=find(dif_top==dif_top_max);
>> real_ind=indminy+dif_top_max_ind-1
>> top_coordinate=[x(min(real_ind)),y(min(real_ind))]
>> indmaxy=find(y==maxy);
>> indminx=find(x==minx);
>> freq_vale_bottom=mode(y(max(indmaxy):max(indminx)));
>> freq_ind_bottom=find(y==freq_vale_bottom);
>> num_bottom=max(freq_ind_bottom)-max(indmaxy)+1
>> new_col=ones(num_bottom,1)*maxy;
>> dif_bottom=new_col-y((max(indmaxy):max(freq_ind_bottom)));
>> dif_bottom_max=max(dif_bottom);
>> dif_bottom_max_ind=find(dif_bottom==dif_bottom_max);
>> real_ind_bottom=max(indmaxy)+min(dif_bottom_max_ind)-1;
>> bottom_coordinate=[x(real_ind_bottom),y(real_ind_bottom)];
```
