# Supplementary material for: Digital image processing method for estimating leaf length and width tested using kiwifruit leaves (Actinidia chinensis Planch)
Source: PLoS One. 2020 Jul 6;15(7):e0235499. doi: 10.1371/journal.pone.0235499 (PMC7337316; doi:10.1371/journal.pone.0235499)
Supplement: S5 Appendix — (PDF) [file pone.0235499.s005.pdf]

Calculating the length and width of kiwifruit leaf

```
>> [mm nn]=size(bw4)
>> ratio=21/mm
>>  length=(((top_coordinate(1)-bottom_coordinate(1))^2+(top_coordinate(2)-bottom_coordinate(2))^2)^0.5)*ratio
>>  width=max(sum(bw4,2))*ratio
```
